# A Linear Birefringence Measurement Method for an Optical Fiber Current Sensor

**DOI:** 10.3390/s17071556

**Published:** 2017-07-03

**Authors:** Shaoyi Xu, Haiming Shao, Chuansheng Li, Fangfang Xing, Yuqiao Wang, Wei Li

**Affiliations:** 1 School of Mechanical and Electrical Engineering, China University of Mining and Technology, Xuzhou 221116, China; xutianyia@126.com (S.X.); shi_xian_zhong@163.com (Y.W.); 2 National Institute of Metrology (NIM), Beijing 100029, China; shaohm@hotmail.com (H.S.); nimlcs@hotmail.com (C.L.); 3 School of Mechatronic Engineering, Xuzhou College of Industrial Technology, Xuzhou 221116, China; cumtxff@cumt.edu.cn

**Keywords:** optical fiber current sensor, linear birefringence, multi-valued problem

## Abstract

In this work, a linear birefringence measurement method is proposed for an optical fiber current sensor (OFCS). First, the optical configuration of the measurement system is presented. Then, the elimination method of the effect of the azimuth angles between the sensing fiber and the two polarizers is demonstrated. Moreover, the relationship of the linear birefringence, the Faraday rotation angle and the final output is determined. On these bases, the multi-valued problem on the linear birefringence is simulated and its solution is illustrated when the linear birefringence is unknown. Finally, the experiments are conducted to prove the feasibility of the proposed method. When the numbers of turns of the sensing fiber in the OFCS are about 15, 19, 23, 27, 31, 35, and 39, the measured linear birefringence obtained by the proposed method are about 1.3577, 1.8425, 2.0983, 2.5914, 2.7891, 3.2003 and 3.5198 rad. Two typical methods provide the references for the proposed method. The proposed method is proven to be suitable for the linear birefringence measurement in the full range without the limitation that the linear birefringence must be smaller than π/2.

## 1. Introduction

An optical fiber current sensor (OFCS) has a number of advantages over the conventional current sensor, such as high precision, high sensitivity, wide dynamic range and immunity to electro-magnetic interference. Thus, it has attracted wide attention in the past three decades. For example, an OFCS has been proposed based on the Fabry-Perot interferometer using a fiber Bragg grating demodulation [1]; an OFCS has been reported based on a long-period fiber grating with a permanent magnet [2]; and an OFCS has been designed based on microfiber and chrome-nickel wire [3]. Moreover, the most widely used OFCS mechanism by far, the Faraday effect, which has been explored in many configurations [4,5,6,7]. In this case, the light propagating in a sensing fiber experiences a rotation in the angle of polarization in the presence of an external magnetic field. The applications of the OFCS using the Faraday effect include the direct current measurement for process control and protection in electrowinning industry [6], the current measurement for revenue metering as well as for the substation control and protection in electric power industry [7], and the current measurement for buried pipelines protection in an urban rail transit system [8,9]. It is well known that the effect of linear birefringence on OFCS is difficult to eliminate, which may degrade the performance of OFCS seriously. Effective elimination methods have been developed based on two types of optical fibers, including the spun high-birefringent optical fiber and the low-birefringent optical fiber. It is proven that the OFCS with the spun high-birefringent optical fiber has a good thermal stability [10,11], however, the high cost of fabrication limits the applications of this optical fiber at present. Moreover, the low-birefringent optical fiber is usually annealed [5], twisted [12] or wound along the designed geometric path [8,13] to minimize the effect of linear birefringence. Among them, it is found that the annealed fiber is easily damaged during the installation process. The twisted optical fiber produces the reciprocal circular birefringence through the shearing stress, which may decrease as time goes on. The sensing fibers wound along the cylindrical [8] and toroidal [13] spiral paths have been proposed based on the geometrical rotation effect. In these sensing fibers, the produced circular birefringence only depends on the geometrical parameters of the path rather than the stress, which provides the effective method to eliminate the linear birefringence in the low-birefringent sensing fiber. In this method, the accurate measurement of the linear birefringence can help to optimize the design of the geometrical path.

The first one of the typical measurement methods of the linear birefringence has been proposed by Ren, who applied the isotropy of circularly polarized light to measure the linear birefringence of the low-birefringent sensing fiber [14]. In this method, the circularly polarized input light can eliminate the effect of the azimuth angle between λ/4 plate and the low-birefringent sensing fiber. An accuracy of 4% in the linear birefringence can be achieved by this method. The second typical measurement method of the linear birefringence is the one proposed by Tentori [15]. In this method, the input polarizer is rotated from 0° to 180°. The polarization state of the output light from the low-birefringent sensing fiber describes a major circle in Poincare sphere. Each point in the major circle can be represented by the Stokes vector [S_0_; S_1_; S_2_; S_3_]. And the linear birefringence can be obtained after determining the maximum or minimum value of the S_3_. The last of the typical measurement methods of the linear birefringence has been proposed by Segura [16]. This method, based on the use of the Faraday effect, and used to measure the linear birefringence with an accuracy of 5% seems to be simple, fast and requires short lengths of fiber. It is noted however that this method needs to fix the transmission axis of the Wollaston prism at 45° with respect to the input polarization. We can find that the three methods described above are all suitable for the measurement of the linear birefringence of the low-birefringent sensing fiber when the linear birefringence is not greater than π/2. If the linear birefringence is greater than π/2, the above methods cannot determine the accurate value of the linear birefringence due to the multi-valued problem that occurs during the measurement. It is known that the linear birefringence of the low-birefringent sensing fiber in an OFCS is usually not smaller than π/2 due to the bending-induced factor. If the OFCS works under varying temperature conditions, the linear birefringence may be greater. Thus, the multi-valued problem may be one of the difficulties to measure the linear birefringence in the OFCS. Moreover, it is known that the principle axes of the low-birefringent sensing fiber are difficult to determine. This may be another difficulty.

In this paper, we propose a linear birefringence measurement method for the OFCS. The configuration of the linear birefringence measurement system is illustrated firstly. The steps of the measurement method are then demonstrated. Among these steps, the elimination method is proposed to eliminate the effect of the azimuth angles between the sensing fiber and the two polarizers. The multi-valued problem of the measurement result is simulated and its solution is presented. Finally, a series of experiments based on the different sensor heads of the OFCS have been conducted to prove the feasibility of our proposed method. When the linear birefringence is not greater than π/2, the typical methods proposed by Ren [14] and Tentori [15] provide the references for our proposed method.

## 2. Linear Birefringence Measurement Method for the OFCS

**Figure 1:**
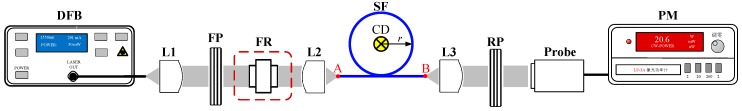
Configuration of the linear birefringence measurement system: L1 to L3 are the aspheric lenses, whose effective focal length is about 11 mm and NA is about 0.25; FP is the fixed polarizer; FR is the Faraday rotator, which is only applied to obtain the *P*_3_ and *P*_4_ shown in Equations (4) and (5); SF is the sensing fiber in the sensor head; CD is the conductor; RP is the rotated polarizer; PM is the power meter.

The configuration of the linear birefringence measurement system is shown in Figure 1, which mainly includes a distributed feedback (DFB) laser diode, a fixed polarizer, three aspheric lenses, a sensor head of the OFCS, a rotated polarizer, and a power meter. The DFB laser diode (model DFB-I-50, LabBang Co. Ltd., Beijing, China) was a central wavelength at 1550 nm and its −20 dB spectral width is about 0.2 nm. Moreover, for the fixed polarizer and rotated polarizer (model LPIREA050-C, Thorlabs Co. Ltd., Newton, NJ, USA), the extinction ratios are both greater than 2000:1 at 1550 nm. Finally, for the sensing fiber (model LB 1550-125, Oxford Electronics Co. Ltd., Hants, UK) in the sensor head, its inherent linear birefringence is 4°/4 m and its bending radius is defined as *r* in this work. During our application in an urban rail transit system, the *r* is usually designed as about 40 cm.

In Figure 1, the light is first generated by the DFB laser diode. The pigtail of the DFB is the single mode fiber, whose mode field diameter is about 10 um. Thus, when the light is collimated by L1, the spot diameter and the divergence angle of the collimated light are estimated at 2.2 mm and 0.052°, respectively. Then, the collimated light passes through the FP to form a linearly polarized light. The linearly polarized light continues to enter into the SF through L2. It is assumed that the principle polarization axes of the SF are *x* axis and *y* axis, respectively. There are the linear birefringence and the Faraday rotation in the SF. Among them, the former one is induced by the refractive index difference between *x*-component and *y*-component while the latter one is induced by the current in the CD. Thus, the output light from the SF is elliptically polarized. The elliptically polarized light is collimated by L3 and is then analyzed by the RP. Finally, the light intensity from the RP is detected by the PM.

In Figure 1, there are two azimuth angles of the transmission axes of the FP and RP with respect to the principle polarization axes of SF, which are formed at the points A and B, respectively. And the azimuth angles are defined as *α* and *β*, respectively. Since the SF in the OFCS is the low-birefringent sensing fiber, the orientations of its principle polarization axes are usually difficult to determine, which causes that the azimuth angles *α* and *β* to be unknown. We believe that the linear birefringence can be measured only after the effect of the azimuth angles *α* and *β* has been eliminated.

**Figure 2:**
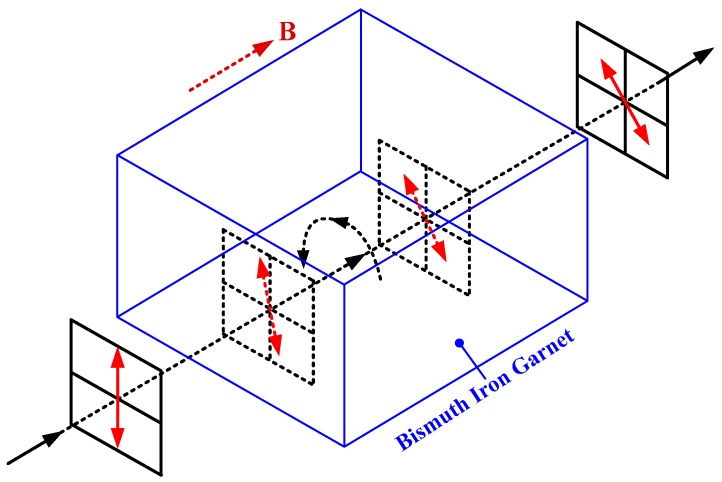
The working mechanism of the Faraday rotator.

In this work, an effective method is proposed to measure the linear birefringence in the SF. It is divided into three steps: first, we keep the transmission axes of the FP and RP parallel, which is the feasible operation using the high-precision rotation mount; then, the effective elimination method on the effect of the azimuth angles *α* and *β* is proposed based on the orthogonal modulation and a Faraday rotator shown in Figure 2; finally, the multi-valued problem on the linear birefringence is solved. The material for the Faraday rotator is bismuth iron garnet. It is noted that the azimuth angles *α* and *β* are the same, which can be both represented by *θ* in this work.

According to the configuration shown in Figure 1, it is assumed that the complex amplitude of the light vector from the FP is *E_in_*. Thus, the complex amplitude of the input light vector of the SF is *E_A_* = [*E_in_*cos*θ*; *E_in_*sin*θ*]. Moreover, the linear birefringence and the Faraday rotation work together on the SF. Thus, the Jones matrix *J_sf_* of the SF can be obtained as Equation (1) [17]:

(1) $$J_{sf}=\left[\begin{array}{cc} \cos\sqrt{F^{2}+{\left(\delta /2\right)}^{2}}+i\left(\delta /2\right)\frac{\sin\sqrt{F^{2}+{\left(\delta /2\right)}^{2}}}{\sqrt{F^{2}+{\left(\delta /2\right)}^{2}}} & -F\frac{\sin\sqrt{F^{2}+{\left(\delta /2\right)}^{2}}}{\sqrt{F^{2}+{\left(\delta /2\right)}^{2}}}\\ F\frac{\sin\sqrt{F^{2}+{\left(\delta /2\right)}^{2}}}{\sqrt{F^{2}+{\left(\delta /2\right)}^{2}}} & \cos\sqrt{F^{2}+{\left(\delta /2\right)}^{2}}-i\left(\delta /2\right)\frac{\sin\sqrt{F^{2}+{\left(\delta /2\right)}^{2}}}{\sqrt{F^{2}+{\left(\delta /2\right)}^{2}}} \end{array}\right],$$

where, *F* is the Faraday rotation angle, *F* = *VNI*; *V* represents the Verdet constant of the SF, *V* = 0.73 urad/A at 1550 nm; *N* is the number of turns in the fiber loop; *I* is the input current in the CD; *δ* is the phase delay induced by the linear birefringence in the SF. The complex amplitude of the output light vector of the SF is *E_B_* = *J_sf_*·*E_A_*. On this basis, the complex amplitude of the light vector from the RP is *E_out_* = *E_Bx_*cos*θ* + *E_By_*sin*θ*. The light intensity from the RP can be derived as Equation (2):

(2) $$P_{1}\approx \left[\frac{1+{\left(\cos2\theta \right)}^{2}+{\left(\sin2\theta \right)}^{2}\cos2\sqrt{F^{2}+{\left(\delta /2\right)}^{2}}}{2}-\frac{F^{2}}{F^{2}+{\left(\delta /2\right)}^{2}}\sin^{2}\sqrt{F^{2}+{\left(\delta /2\right)}^{2}}{\left(\cos2\theta \right)}^{2}\right]\times E_{in}^{2}.$$

Then, the orientation of the FP remains the same. The RP is subjected to a 90° counter-clockwise rotation with respect to its initial orientation. Thus, the azimuth angle of the transmission axis of the RP with respect to the *x* axis of the SF is changed to (*θ* + π/2). The light intensity from the RP can be derived as:

(3) $$P_{2}\approx \left[\frac{1-{\left(\cos2\theta \right)}^{2}-{\left(\sin2\theta \right)}^{2}\cos2\sqrt{F^{2}+{\left(\delta /2\right)}^{2}}}{2}+\frac{F^{2}}{F^{2}+{\left(\delta /2\right)}^{2}}\sin^{2}\sqrt{F^{2}+{\left(\delta /2\right)}^{2}}{\left(\cos2\theta \right)}^{2}\right]\times E_{in}^{2}.$$

Moreover, the Faraday rotator is installed between the FP and the L2 to rotate the output polarization state from the FP by 45° counterclockwise. With the help of the Faraday rotator, the azimuth angle of the transmission axis of the FP with respect to the *x* axis of the SF is changed to *θ* + π/4 and the azimuth angle of the RP is changed as *θ* + π/4. In this case, the light intensity from the RP can be derived as:

(4) $$P_{3}\approx \left[\frac{1+{\left(\sin2\theta \right)}^{2}+{\left(\cos2\theta \right)}^{2}\cos2\sqrt{F^{2}+{\left(\delta /2\right)}^{2}}}{2}-\frac{F^{2}}{F^{2}+{\left(\delta /2\right)}^{2}}\sin^{2}\sqrt{F^{2}+{\left(\delta /2\right)}^{2}}{\left(\sin2\theta \right)}^{2}\right]\times E_{in}^{2}.$$

Finally, the azimuth angle of the FP keeps *θ* + π/4 using the Faraday rotator. The azimuth angle of the RP is changed to (*θ* + 3π/4). In this case, the light intensity from the RP can be derived as:

(5) $$P_{4}\approx \left[\frac{1-{\left(\sin2\theta \right)}^{2}-{\left(\cos2\theta \right)}^{2}\cos2\sqrt{F^{2}+{\left(\delta /2\right)}^{2}}}{2}+\frac{F^{2}}{F^{2}+{\left(\delta /2\right)}^{2}}\sin^{2}\sqrt{F^{2}+{\left(\delta /2\right)}^{2}}{\left(\sin2\theta \right)}^{2}\right]\times E_{in}^{2}.$$

According to Equations (2) and (3), the heterodyne method is applied to process the light intensities *P*_1_ and *P*_2_. The first heterodyne (*W*_1_) output is given as Equation (6). Then the heterodyne method goes on to process the light intensities *P*_3_ and *P*_4_. The second heterodyne (*W*_2_) output is given as Equation (7). Finally, the sum of the two heterodyne outputs can be obtained as Equation (8):

(6) $$W_{1}=\frac{P_{1}-P_{2}}{P_{1}+P_{2}}={\left(\cos2\theta \right)}^{2}+{\left(\sin2\theta \right)}^{2}\cos2\sqrt{F^{2}+{\left(\delta /2\right)}^{2}}-2\frac{F^{2}}{F^{2}+{\left(\delta /2\right)}^{2}}\sin^{2}\sqrt{F^{2}+{\left(\delta /2\right)}^{2}}{\left(\cos2\theta \right)}^{2}.$$

(7) $$W_{2}=\frac{P_{3}-P_{4}}{P_{3}+P_{4}}={\left(\sin2\theta \right)}^{2}+{\left(\cos2\theta \right)}^{2}\cos2\sqrt{F^{2}+{\left(\delta /2\right)}^{2}}-2\frac{F^{2}}{F^{2}+{\left(\delta /2\right)}^{2}}\sin^{2}\sqrt{F^{2}+{\left(\delta /2\right)}^{2}}{\left(\sin2\theta \right)}^{2}.$$

(8) $$U=W_{1}+W_{2}=1+\cos2\sqrt{F^{2}+{\left(\delta /2\right)}^{2}}-2\frac{F^{2}}{F^{2}+{\left(\delta /2\right)}^{2}}\sin^{2}\sqrt{F^{2}+{\left(\delta /2\right)}^{2}}.$$

According to Equation (8), the relationship of the *F*, the *δ* and the U can be obtained as:

(9) $$\frac{2F^{2}+{\left(\delta /2\right)}^{2}}{F^{2}+{\left(\delta /2\right)}^{2}}\sin^{2}\sqrt{F^{2}+{\left(\delta /2\right)}^{2}}-\left(1-\frac{U}{2}\right)=0.$$

In Equation (9), the *F* and the U are known while the *δ* is unknown. We define the function *f*(*δ*) as:

(10) $$f\left(\delta \right)=\frac{2F^{2}+{\left(\delta /2\right)}^{2}}{F^{2}+{\left(\delta /2\right)}^{2}}\sin^{2}\sqrt{F^{2}+{\left(\delta /2\right)}^{2}}-\left(1-\frac{U}{2}\right).$$

**Figure 3:**
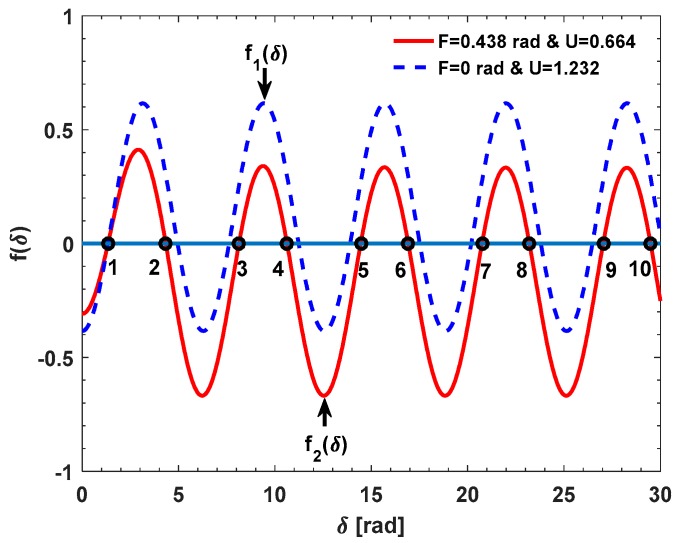
The {*f_i_*(*δ*)} versus the *δ* in the range of 0–30 rad: *F*_1_ = 0 rad and U_1_ = 1.232 while *F*_2_ = 0.438 rad and U_2_ = 0.664.

There may be a multi-valued problem to determine *δ* during the process of solving the equation *f*(*δ*) = 0. We conduct two simulations to demonstrate this multi-valued problem. In the first simulation, the *N* is set as 15 and the input current is set as 40,000 A, which leads to *F* being about 0.438 rad. The theoretical value of *δ* is about 1.337 rad (<π/2 rad) [18]. On these bases, the U is about 0.664 based on Equation (8). The *f*(*δ*) versus the *δ* in the range of 0–30 rad is shown as the red curve in Figure 3. 

**Figure 4:**
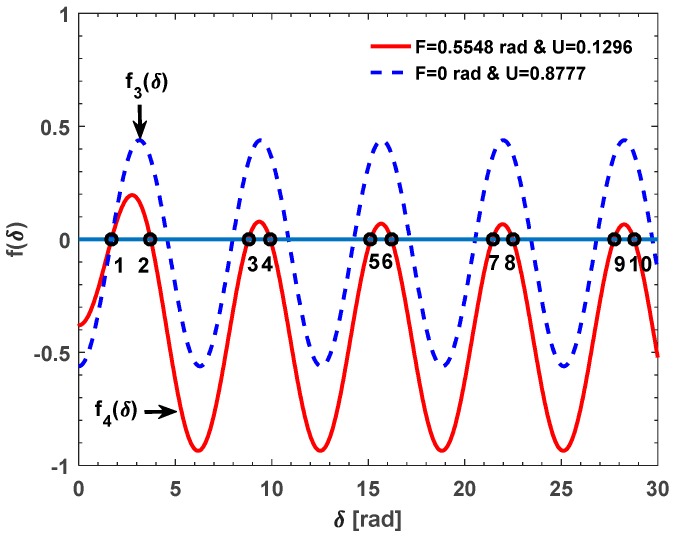
The {*f_i_*(*δ*)} versus the *δ* in the range of 0–30 rad: *F*_3_ = 0 rad and U_3_ = 0.8777 while *F*_4_ = 0.5548 rad and U_4_ = 0.1296.

We can find that there are ten solutions to the equation *f*(*δ*) = 0, including the exact solution *δ* = 1.337 rad (point 1). Given that the *F* and the U are changed as the input current while the *δ* is unchanged, we believe that the *δ* can be solved based on the datasets {*F_i_*} and {U*_i_*} obtained in the different input current. For example, when the input current is 0 and 40,000 A, the *F* is about 0 and 0.438 rad. Thus, the U is about 1.232 and 0.664, respectively. The corresponding functions are defined as *f*_1_(*δ*) and *f*_2_(*δ*), respectively. The {*f_i_*(*δ*)} versus *δ* plots are shown in Figure 3. We can find that *f*_1_(*δ*) and *f*_2_(*δ*) are all equal to zero only when the *δ* is equal to 1.337 rad (point 1). The other nine solutions (point 2 to 10) of *f*_2_(*δ*) = 0 are not the solutions of *f*_1_(*δ*) = 0, which are shown in Figure 3. In the second simulation, the *N* is set as 19 and the theoretical value of the *δ* is about 1.693 rad (>π/2 rad) [18]. When the input current is about 0 and 40,000 A, *F* is about 0 and 0.5548 rad. Thus, the U is about 0.8777 and 0.1296, respectively. Similarly, the corresponding functions are defined as *f*_3_(*δ*) and *f*_4_(*δ*), respectively. The {*f_i_*(*δ*)} versus *δ* curves are shown in Figure 4. Obviously, *f*_3_(*δ*) and *f*_4_(*δ*) are all equal to zero only when the *δ* is equal to 1.693 rad (point 1). The other nine solutions (point 2 to 10) of *f*_3_(*δ*) = 0 are not the solutions of *f*_4_(*δ*) = 0, which are shown in Figure 4.

Thus, according to the above simulation results, for solving the multi-valued *δ* problem, the datasets {*F_i_*} and {U*_i_*} are firstly obtained based on the different values of the input current. On this basis, the sets of equations {*f_i_*(*δ*) = 0} can be obtained as Equation (11).

(11) $$\left\{ \begin{array}{c} \frac{2{F_{1}}^{2}+{\left(\delta /2\right)}^{2}}{{F_{1}}^{2}+{\left(\delta /2\right)}^{2}}\sin^{2}\sqrt{{F_{1}}^{2}+{\left(\delta /2\right)}^{2}}-\left(1-\frac{U_{1}}{2}\right)=0\\ \frac{2{F_{2}}^{2}+{\left(\delta /2\right)}^{2}}{{F_{2}}^{2}+{\left(\delta /2\right)}^{2}}\sin^{2}\sqrt{{F_{2}}^{2}+{\left(\delta /2\right)}^{2}}-\left(1-\frac{U_{2}}{2}\right)=0\\ \vdots \\ \frac{2{F_{k}}^{2}+{\left(\delta /2\right)}^{2}}{{F_{k}}^{2}+{\left(\delta /2\right)}^{2}}\sin^{2}\sqrt{{F_{k}}^{2}+{\left(\delta /2\right)}^{2}}-\left(1-\frac{U_{k}}{2}\right)=0 \end{array}\right..$$

In this work, the effective solution of {*f_i_*(*δ*) = 0} has been proposed based on the newer version of Matlab software (e.g., Matlab R2016a). It includes that: first, the particle swarm optimization is applied to obtain the local optimal results of the *δ*; then, the ‘Multistart’ solver in the global optimization toolbox is run from the random initial points that come from the local optimal results. In this step, the ‘lsqnonlin’ solver starts each random initial point and finds a minimum of the sum of squares of the functions described in {*f_i_*(*δ*)}. Finally, the minimum of the sum of squares in all initial points is searched and the corresponding *δ* is the desirable result.

## 3. Experimental

**Figure 5:**
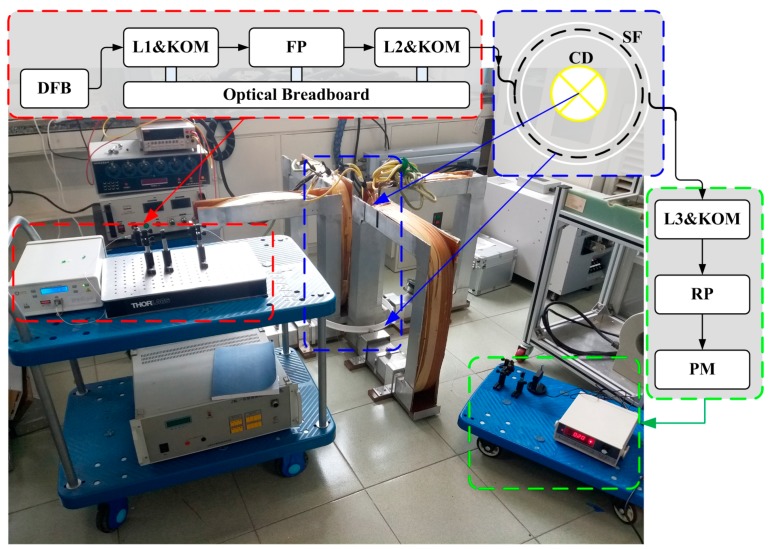
The physical map and its illustration of the experimental system.

In order to verify the feasibility of our proposed method, a series of experiments are conducted based on the different sensor heads of our OFCS. What these sensors all have in common is that their diameters are all 40 cm. Referring to the configuration shown in Figure 1, the magnetic field is produced directly by the input current in the conductor, which requires a strong current generator. In this experiment, the current generator is substituted by a magnetic field generator that can produce the equivalent magnetic field of a maximum of 50,000 A. Moreover, an optical breadboard and a five-axis kinematic optic mount (KOM) are very useful for the output light from the FP to enter into the sensing fiber. The physical map and its illustration of the experimental system are shown in Figure 5. Finally, it is noted that there are two input current during the experiments, one is 0 A and the other is 40,000 A.

In the first experiment, the number of turns of the sensing fiber in the sensor head is about 15. When the input current is 0 A, the four light intensities *P*_1_ to *P*_4_ shown in Equations (2) to (5) are 11.1, 5.6, 14.69 and 1.76 mW, respectively, which are all detected by the PM. The heterodyne outputs *W*_1_ and *W*_2_ are about 0.3293 and 0.7860. The sum U is about 1.1154. Moreover, the Faraday rotation angle *F* is about 0. When the input current is 40,000 A, the *P*_1_ to *P*_4_ are 10.5, 7.18, 12.97 and 3.42 mW, respectively. The *W*_1_ and *W*_2_ are about 0.1878 and 0.5827. The U is about 0.7705. And the *F* is about 0.438 rad. Thus, the equations in the first experiment can be obtained as:

(12) $$\left\{ \begin{array}{l} \sin^{2}\left(\delta /2\right)-0.4423=0\\ \frac{0.3837+{\left(\delta /2\right)}^{2}}{0.1918+{\left(\delta /2\right)}^{2}}\sin^{2}\sqrt{0.1918+{\left(\delta /2\right)}^{2}}-0.6148=0 \end{array}\right..$$

**Figure 6:**
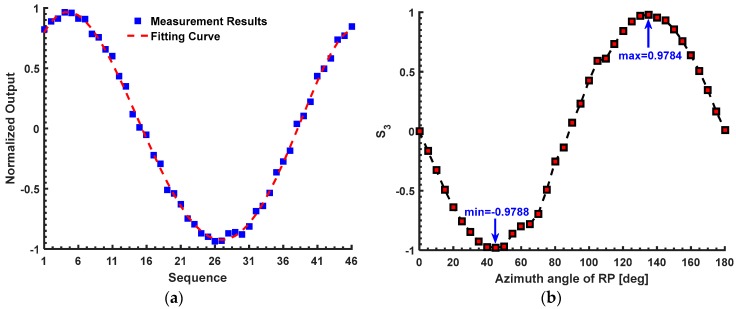
The measurement result of the reference methods in the first experiment: (**a**) the method proposed by Ren; (**b**) the method proposed by Tentori.

Thus, after solving the Equation (12), we can get the linear birefringence *δ* about 1.3577 rad in the first experiment, which is slightly larger than the theoretical value (*δ* = 1.337 rad) shown in the first simulation. In the first simulation, we only consider the bending-induced birefringence and the inherent linear birefringence. However, in the first experiment, there may be a small amount of the extra linear birefringence that is produced during the winding of the sensing fiber. Thus, we believe this measurement result is right. As mentioned in the Introduction section, the measurement methods proposed by Ren [14] and Tentori [15] are both effective for the case that the *δ* is not larger than π/2 rad (about 1.5708 rad). They can provide the references for our proposed method in the first experiment. According to [14], the first reference method proposed by Ren requires a λ/4 plate (model WPQ05M-1550, Thorlabs Co. Ltd., Newton, NJ, USA) and a Wollaston prism (model WP10, Thorlabs Co. Ltd., Newton, NJ, USA). For the sensor head in the first experiment, the measurement process is shown in Figure 6a. The measurement result of the first reference method is about 0.9763, which is equal to sin(*δ*). Thus, the value of *δ* is about 1.3526 rad, which is approximately consistent with the measurement result of our proposed method (*δ* = 1.3577 rad). Moreover, the second reference method proposed by Tentori [15] is also used to measure the *δ* of the sensor head in the first experiment. Compared with our configuration shown in Figure 1, in the second reference method, the FP is replaced by a RP and the output light from SF is detected directly by a polarimeter (model DOP-101D, General Photonics Co. Ltd., Chino, CA, USA). It is assumed that the output light from the SF can be expressed by the Stokes vector [S_0_; S_1_; S_2_; S_3_]. Among them, the S_3_ can be expressed as –sin 2*α*sin *δ*, where the α denotes the multi-parameter model, including the azimuth angle of the transmission axes of the RP with respect to the principle polarization axes of SF. Since the sin 2α is in the range from −1 to 1, the –sin 2*α*sin *δ* can reach its maximum sin *δ* or minimum –sin *δ* after rotating the RP. For the sensor head in the first experiment, the measurement result of the second reference method is shown in Figure 6b. It can be found that the minimum S_3_ is about −0.979. Thus, the *δ* is about 1.3655 rad, which is also approximately consistent with the measurement results of our proposed method (*δ* = 1.3577 rad) and the method proposed by Ren (*δ* = 1.3526 rad).

In the second experiment, the number of turns of the sensing fiber in the sensor head is about 19. When the input current is 0 A, the *P*_1_ to *P*_4_ are 9.23, 7.9, 13.46 and 2.93 mW. The *W*_1_ and *W*_2_ are about 0.0776 and 0.6425. The U is about 0.7201. The *F* is about 0. When the input current is 40,000A, the *P*_1_ to *P*_4_ are 6.64, 10.49, 10.18 and 5.68 mW, respectively. The *W*_1_ and *W*_2_ are about −0.2248 and 0.2837. The U is about 0.059. And the *F* is about 0.5548 rad. Thus, the equations in the second experiment can be obtained as:

(13) $$\left\{ \begin{array}{l} \sin^{2}\left(\delta /2\right)-0.6399=0\\ \frac{0.6156+{\left(\delta /2\right)}^{2}}{0.3078+{\left(\delta /2\right)}^{2}}\sin^{2}\sqrt{0.3078+{\left(\delta /2\right)}^{2}}-0.9705=0 \end{array}\right..$$

**Figure 7:**
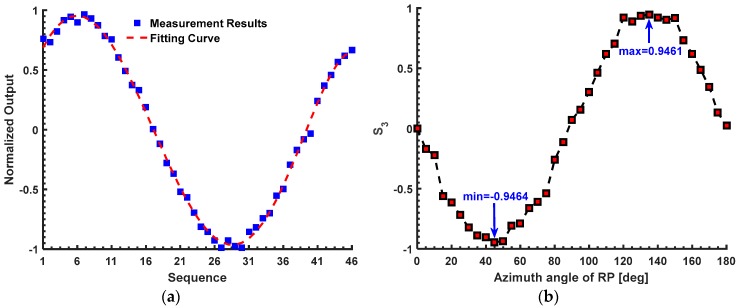
The measurement result of the reference methods in the second experiment: (**a**) the method proposed by Ren; (**b**) the method proposed by Tentori.

Thus, after solving Equation (13), we can get that the linear birefringence *δ* is about 1.8425 rad for the sensor head in the second experiment. It is noted that there is the multi-valued problem for the reference methods proposed by Ren [14] and Tentori [15] when the *δ* is larger than π/2 rad. With regard to the sensor head in the second experiment, the measured sin *δ* using the two reference methods are about 0.9693 and 0.946, which are shown in Figure 7. Obviously, it cannot determine the accurate value of the *δ* only based on the sin *δ*. Thus, the accuracy of our proposed method has to be evaluated indirectly based on the sin *δ* in the second experiment. The sin *δ* is about 0.9633 using our proposed method, which is approximately consistent with the results obtained by the two reference methods (sin *δ =* 0.9693 and sin *δ =* 0.946). Moreover, the *δ* in the second experiment (*δ* = 1.8425 rad) is really larger than the first experiment (*δ* = 1.3577 rad) with the increasing the number of turns of the sensing fiber.

We continue to test the linear birefringence of the different sensor heads. Among them, the numbers of turns of the sensing fiber are 23, 27, 31, 35 and 39, respectively. It is rare that the number of turns (*N*) is greater than 40 in our practical application of the stray current measurement [9]. Thus, the sensor head with *N* ≥ 40 is not tested in this work. We can find that the *δ* obtained by our proposed method is about 2.0983, 2.5914, 2.7891, 3.2003 and 3.5198 rad when the *N* is about 23, 27, 31, 35 and 39. The *δ* is increased with the increase of the *N*. Since the sensing fiber is manually wound in the sensor head, the linear birefringence produced in the manual operation cannot be the same for each sensor head.

**Figure 8:**
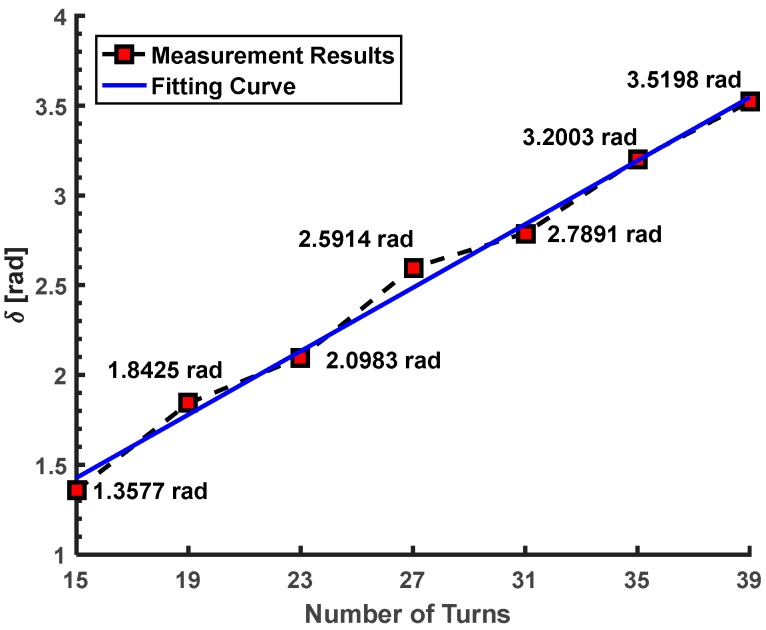
The measurement results of *δ* when *N* is about 15, 19, 23, 27, 31, 35 and 39, respectively.

Thus, this increase is close to linear, which is shown in Figure 8. Moreover, these sensor heads are all measured by the two reference methods proposed by Ren [14] and Tentori [15]. In the reference method proposed by Ren [14], the results (sin *δ*) are about 0.8754, 0.5341, 0.3049, −0.0511 and −0.357 when the *N* is about 23, 27, 31, 35 and 39. In the reference method proposed by Tentori [15], the results (sin *δ*) are about 0.8616, 0.5091, 0.4161, −0.0657 and −0.3744 when the *N* is about 23, 27, 31, 35 and 39. The results (sin *δ*) obtained by our proposed method are about 0.8641, 0.5229, 0.3452, −0.0587 and −0.3693 when the *N* is about 23, 27, 31, 35 and 39. Similarly with the first and second experiments, the results obtained by these two reference methods can prove the feasibility of our proposed method indirectly.

## 4. Conclusions

In this paper, we propose a linear birefringence measurement method for an OFCS. First, the optical configuration of the measurement system is presented. Then, the elimination method of the effect of the azimuth angles between the sensing fiber and the two polarizers is demonstrated based on the orthogonal modulation and a Faraday rotator. The relationship of the linear birefringence, the Faraday rotation angle and the final output is determined based on the Jones matrix calculus and the heterodyne method. Moreover, the multi-valued problem on the linear birefringence is simulated and its solution is illustrated when the linear birefringence is unknown. Finally, the experiments are conducted to prove the feasibility of the proposed method. When the numbers of turns of the sensing fiber in the OFCS are about 15, 19, 23, 27, 31, 35, and 39, the measured linear birefringence obtained by the proposed method are about 1.3577, 1.8425, 2.0983, 2.5914, 2.7891, 3.2003 and 3.5198 rad. Two typical methods provide the references for the proposed method. According to the comparison results, we find that the proposed method is suitable for the linear birefringence measurement in the full range without the limitation that the linear birefringence must be smaller than π/2. In this work, we only test the bending induced and inherent linear birefringence, which have been used to verify the feasibility of our proposed method. It is known that there is no accurate theory to express the temperature induced linear birefringence. This linear birefringence is absolutely unknown. Thus, in the next work, we will continue to test the temperature induced linear birefringence in the OFCS based on our proposed method, which will very useful for the suppression of the temperature error in OFCS.
